# Clinical characteristics of pyoderma gangrenosum: Case series and literature review

**DOI:** 10.1097/MD.0000000000039634

**Published:** 2024-09-13

**Authors:** Rina Su, Yaqi Tan, Shiguang Peng

**Affiliations:** a Department of Dermatology, Beijing Chao-yang Hospital, Capital Medical University, Beijing, China.

**Keywords:** clinical characteristics, comorbidities, corticosteroids, immunosuppressants, pyoderma gangrenosum

## Abstract

**Background:**

Pyoderma gangrenosum (PG) is a neutrophilic skin disease characterized by recurrent painful cutaneous ulcers, often accompanied by inflammatory bowel disease, joint pain, and other systemic damage. This disease is relatively rare in clinical practice and its diagnosis and treatment are often delayed, leading to secondary infections in the skin lesions, prolonged disease course, and increased disease burden on patients. This study retrospectively analyzed the clinical characteristics and treatment strategies of patients with PG admitted to our hospital and conducted a literature review, in order to improve the understanding of the disease among clinical doctors, enable patients to receive better diagnosis and treatment, and ultimately improve patient prognosis.

**Methods:**

Clinical data of patients diagnosed with PG and hospitalized in Beijing Chaoyang Hospital, Capital Medical University from January 2014 to December 2022 were retrospectively collected. The clinical manifestations, treatment strategies, efficacy, and disease outcomes were analyzed.

**Results:**

A total of 14 patients, including 8 males and 6 females, aged 14 to 66 years, were included. Skin lesion types: 13 cases were ulcer-type, 1 case was pustule combined with ulcer-type, and the lower limbs were the most commonly affected areas. All the 14 patients presented with comorbidities. All patients were treated with glucocorticoids, with a daily dose equivalent to 20 to 100 mg prednisone and a median dose of 40 mg. Among them, 3 patients were treated with minocycline in combination, 1 patient was treated with mycophenolate mofetil 0.5 twice daily in combination, 1 patient was treated with cyclophosphamide 0.1 once daily in combination, and 1 patient was treated with thalidomide 0.1 every night in combination.

**Conclusion:**

PG is a relatively rare immune-related skin disease. Our small sample data analysis found that male PG is not uncommon in the Chinese population. Systemic glucocorticoids can quickly control the symptoms of PG in most patients with PG. In patients with poor efficacy or limited use of glucocorticoids, immunosuppressive drugs or novel targeted drugs such as biologics or small-molecule drugs should be used in combination as early as possible. Skin lesion care focuses on preventing infection, avoiding surgical debridement, and emphasizing pain management and the symptomatic treatment of comorbidities.

## 1. Introduction

Pyoderma gangrenosum (PG) is a relatively uncommon neutrophilic dermatological condition, primarily characterized by the rapid development of painful ulcerative lesions with notable border disruption and surrounding erythema.^[1]^ Epidemiological data indicate a mean age of onset in the mid-40s, with incidence rates being relatively low, estimated at several cases per million individuals annually. There is a recognized association between PG and various immune-mediated disorders, with inflammatory bowel disease (IBD) and rheumatoid arthritis (RA) being among the most prevalent comorbidities. The underlying etiology of PG remains elusive; however, it is widely regarded as an autoinflammatory disorder. Management strategies for PG predominantly involve immunosuppressive therapy, which is often complemented by meticulous wound care to address cutaneous manifestations. In pursuit of a more nuanced understanding of the clinical spectrum of PG, the present study undertook a retrospective analysis of clinical data from 14 patients diagnosed with PG. The findings from this analysis provide valuable insights into the heterogeneity of clinical presentations and outcomes associated with this challenging dermatosis.

## 2. Materials and methods

### 2.1. Clinical data

All procedures performed in studies involving human participants were in accordance with the ethical standards of the institutional and/or national research committee and with the 1964 Helsinki Declaration and its later amendments or comparable ethical standards. All patients or relatives had provided informed consent for publication of the case. A retrospective collection of clinical data was conducted on patients diagnosed with PG during their hospital stay at Beijing Chaoyang Hospital, affiliated with Capital Medical University, from January 2014 to December 2022. Patients were diagnosed with PG after a comprehensive evaluation of their clinical manifestations and histopathological characteristics while ruling out other ulcerative skin diseases. This study aimed to enhance the understanding of this condition and improve patient outcomes through a detailed analysis and evidence-based practice.^[2]^

### 2.2. Statistical analysis

Patient data were analyzed using SPSS Version 28.0 (IBM Corp, Armonk, NY) statistical software. For quantitative data, the median, mean, and standard deviation were employed as measures of the central tendency and dispersion. For categorical data, the chi-squared (*χ*^2^) test was used to examine the relationships between the variables. The analysis was conducted in a systematic and rigorous manner to ensure the accuracy and reliability of the results obtained through the application of this advanced statistical tool.

## 3. Results

### 3.1. General clinical data

The analysis included a total of 14 patients, consisting of 8 males and 6 females. The age range of the participants was between 14 and 66 years, with an average age of 46.1 ± 15.8 years and a median age of 47.5 years. The duration of the condition among these individuals varied from 2 to 84 months, with an average duration of 35.8 ± 25.9 months and a median duration of 7 months.

### 3.2. Clinical characteristics

**Figure 1.**
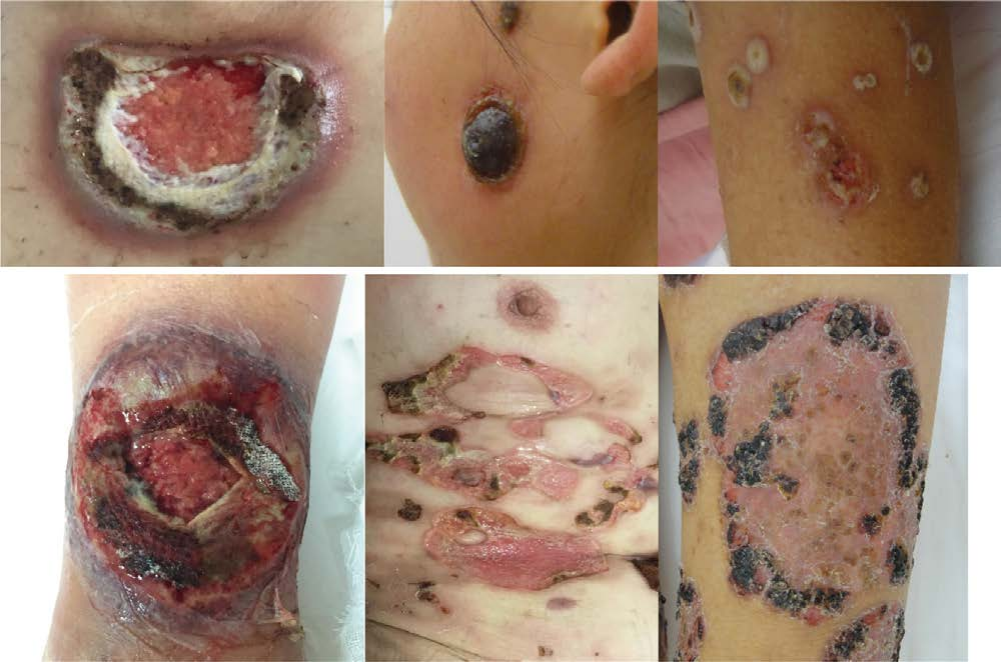
Skin lesion manifestations of patients. All photos were originally taken by the authors and authorized for publication by the patients.

The distribution of skin lesions in the 14 patients studied was predominantly in the lower limbs. Among them, 1 patient involved the face, trunk, and limbs at the same time, 1 patient involved both the trunk and limbs, 2 patients involved both the trunk and lower limbs, 3 patients only involved the limbs, and 7 patients only involved the lower limbs (Fig. 1). In terms of lesion types, 13 cases were ulcerative type, and 1 was pustular combined with ulcerative type.

In regards to comorbidities, within this patient group, 4 presented with anemia, 3 with diabetes, 3 with hypertension, 2 with ulcerative colitis, 2 with multiple myeloma, 2 with cerebral infarction, 2 with heart failure, 2 with tuberculosis, and 2 with hypokalemia. Additionally, there was 1 case each of hyperlipidemia, coronary atherosclerotic heart disease, ischemic hypoxic encephalopathy, pulmonary arterial hypertension, idiopathic pulmonary fibrosis, liver failure, renal failure, hepatitis B virus infection, hypocalcemia, and colon polyps. One patient experienced secondary mercury poisoning due to the use of an unorthodox remedy (Table 1).

**Table 1:** Clinical characteristics of 14 patients with pyoderma gangrenosum.

No	Gender	Age	Disease duration/mo	Skin lesions	Comorbidities	Systemic treatment plan	Outcome
1	Male	61	8	Lower limbs	Hypertension, cerebral infarction	Hydrocortisone 200 mg qd, minocycline 100 mg bid	Got better
2	Male	14	12	Limbs	Mercury poisoning, iron deficiency anemia	Methylprednisolone 32 mg qd	Got better
3	Male	43	9	Abdomen, lower limbs	Ulcerative colitis, hepatitis B	Methylprednisolone 20 mg qd	Got better
4	Male	33	3	Lower limbs	Multiple myeloma, hypokalemia	Methylprednisolone 60 mg qd, CTX 0.1 qd	Got better
5	Male	46	4	Lower limbs	Hyperlipidemia, colon polyps	Prednisone 20 mg bid	Got better
6	Male	49	4	Lower limbs	Cerebral infarction, hypoxic-ischemic encephalopathy, severe anemia, tuberculosis, diabetes	Prednisone 30 mg qd	Dead
7	Male	59	6	Lower limbs	Multiple myeloma	Methylprednisolone 32 mg qd	Got better
8	Male	35	8	Lower limbs	Coronary atherosclerotic heart disease, hypertension, renal insufficiency, pulmonary hypertension, diabetes mellitus	Prednisone 40 mg qd	Got better
9	Female	52	60	Lower limbs, chest	Nutritional chronic anemia, old tuberculous meningitis	Methylprednisolone 40 mg qd, mycophenolate mofetil 0.5 bid	Got better
10	Female	66	48	Back, abdomen, limbs, vulva	Diabetes	Methylprednisolone 40 mg qd + prednisone 20 mg qd, minocycline 100 mg bid	Got better
11	Female	41	3	Limbs	Ulcerative colitis, anemia, hypokalemia, hypocalcemia	Methylprednisolone 40 mg, thalidomide 100 mg qn	Got better
12	Female	23	84	Limbs, trunk	Cardiac insufficiency	Methylprednisolone 30 mg qd, minocycline 100 mg bid	Got better
13	Female	65	5	Lower limbs	Idiopathic pulmonary fibrosis, cardiac insufficiency, hepatic insufficiency	Methylprednisolone 80 mg qd	Got better
14	Female	59	2	Limbs	Hypertension	Prednisone 20 mg qd	Got better

Bid = twice daily, Qd = once daily, Qn = every night.

### 3.3. Laboratory findings

Laboratory tests were conducted in 14 patients during the initial phase of PG treatment. It was observed that 6 patients exhibited an elevation in peripheral white blood cell count, ranging from 12.45 to 17.30 × 10^9^/L with a median value of 15.49 × 10^9^/L, which is above our institution’s normal range of 3.5 to 9.5 × 10^9^/L. Additionally, 5 patients showed a significant increase in erythrocyte sedimentation rate, ranging from 50 to 120 mm/h with a median value of 67 mm/h, surpassing the normal range of our institution, which is 2 to 15 mm/h. Five patients had elevated C-reactive protein levels ranging from 1.33 to 12.7 mg/dL with a median value of 6.9 mg/dL, while the normal range was 0 to 0.8 mg/dL. Furthermore, 5 patients underwent antineutrophil cytoplasmic antibodies (ANCA) examination, of which 1 patient was weakly positive for PR3 antibody, while the rest of the patients were negative.

### 3.4. Histopathological findings

**Figure 2.**
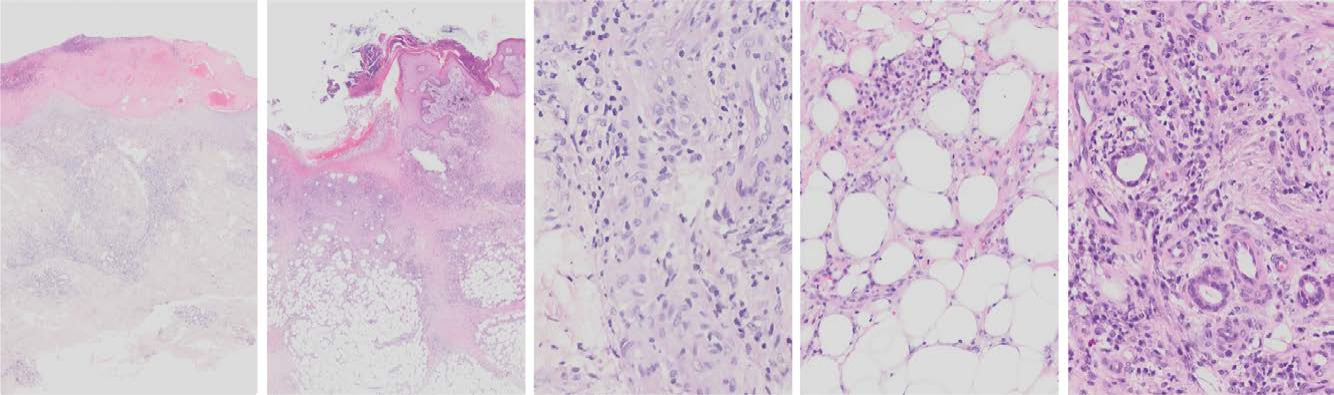
The histopathological manifestations of patients.

Tissue biopsies from the affected skin areas were performed in all patients (Fig. 2). Hematoxylin and eosin (H&E) staining consistently revealed dilated capillaries within the dermis, with some vessels exhibiting necrotic walls. There was a diffuse infiltration of inflammatory cells, predominantly neutrophils and lymphocytes.

### 3.5. Treatment and prognosis

All 14 patients were treated with systemic corticosteroids, with daily dosages equivalent to prednisone ranging from 20 to 100 mg and a median dose of 40 mg. In addition to corticosteroids, 3 patients received concurrent treatment with minocycline, 1 patient was cotreated with mycophenolate mofetil (0.5 mg) twice daily (bid), another with cyclophosphamide at a dosage of 0.1 mg once daily (qd), and 1 patient was administered thalidomide at a dosage of 0.1 mg every night (qn), as detailed in Table 1. Symptomatic treatments for comorbid conditions were administered along with supportive care to prevent adverse reactions related to steroid use. All patients underwent regular wound dressing changes to prevent secondary bacterial infections and promote wound healing. Following treatment, there was an improvement in the skin lesions in all patients. However, during the follow-up period, 1 patient experienced a large cerebral infarction and subsequently died.

## 4. Discussion

PG is a relatively rare condition that presents challenges in both diagnosis and epidemiological research owing to its infrequent occurrence in clinical settings. The incidence rates reported in the literature vary, reflecting the rarity and diagnostic complexity of PG. A population-based study in the United States that employed a validation algorithm and analyzed data from the Explorys platform identified 1971 cases of PG among a cohort of over 31 million adults, yielding an incidence rate of approximately 58 cases per million adults.^[3]^ In contrast, a study from the United Kingdom reported an incidence of approximately 6 cases per million person-years.^[4]^ Similarly, research conducted in Italy found an annual incidence rate of 5.17 new cases per million population.^[5]^ These studies indicate that while PG is uncommon, it does occur with varying frequencies across different populations, and further research is necessary to understand its epidemiology and improve diagnostic accuracy.

Despite the possibility of PG manifesting across all age groups, research has consistently indicated a higher prevalence among older individuals, typically around the age of 50 years. For instance, a cohort study utilizing the UK General Practice Research Database revealed that the median age of patients with PG was 59 years.^[4]^ Similarly, a study based in the United States reported that nearly 70% of patients with PG were aged 50 years or above.^[3]^ Another American study pinpointed the average age of onset for PG at 44.6 years, with studies from Italy and Switzerland producing comparable estimates.^[5,6]^ These findings underscore the significance of age as a factor in the incidence of PG, although this condition can occur at any age. This article presents an analysis of 14 patients diagnosed with PG at our institution. At the time of consultation, the median age was 47.5 years, and the median duration of the disease was 7 months. The age of onset for these patients was consistent with the results of previous international studies.

Most research reports indicate that the prevalence and incidence of PG are slightly higher in women than in men, accounting for approximately 59 to 68% of cases. This article includes an analysis of 14 cases of PG treated at our institution, with males representing 57.14% and females 42.86% of the cases. The proportion of male patients exceeded the figures reported in international studies, suggesting that the incidence rate of PG among males may be higher in the Chinese population. However, this hypothesis requires validation through statistical analysis using a larger sample size.

PG is recognized as a condition with significant morbidity and, as recent studies indicate, is associated with increased mortality rates. Research leveraging data from the UK General Practice Research Database has illuminated the concerning statistic that the mortality rate for patients diagnosed with PG is threefold higher than that of an age- and sex-matched control group.^[4]^ This elevation in mortality persists even when patients with PG are juxtaposed with individuals suffering from other inflammatory conditions, underscoring the seriousness of PG as a health concern. Notably, within the context of this study, there was an account of a patient with PG who experienced a fatal outcome during the follow-up period. The implications of these findings are critical for healthcare providers, highlighting the need for diligent monitoring and comprehensive management strategies for patients with this disease.

PG is typically characterized by rapidly evolving painful ulcers that expand with an erosive edge. The margins of these ulcers often appear red or purple with irregular borders, and erythema surrounding the lesion is frequently observed. PG can manifest as a solitary occurrence, often at sites of trauma, or as multiple new lesions that emerge simultaneously. Patients with PG may experience a chronic, relapsing, or self-limiting disease course. The clinical presentation of PG is diverse, with classifications typically based on the morphology and characteristics of the lesions. The most common form is ulcerative PG, which is characterized by rapidly progressing painful ulcers with erosive changes and surrounding erythema. Bullous PG is primarily characterized by blister formation, which may quickly evolve into ulcers. Pustular PG is characterized by the presence of pustules that can form on the skin or at the edges of an ulcer. Vegetative PG is characterized by proliferative skin changes that may present as a granulomatous or wart-like growth. Peristomal PG occurs around a stoma and typically manifests in the peristomal area. postsurgical PG arises postoperatively, often near surgical incisions. Drug-induced PG is triggered by medications, such as tumor necrosis factor (TNF) inhibitors. Cases that do not conform to any of the aforementioned types are classified as atypical PG, which may present as single or multiple lesions with varied clinical manifestations. In the cohort discussed in this text, 14 patients were included, with 13 presenting with ulcerative PG and 1 with a pustular-ulcerative combination, where pustules rapidly ruptured to form ulcers during the course of the disease.

According to the literature, PG is associated with a variety of immune-mediated diseases in 33% to 56.8% of cases, with IBD being the most common. Other associated conditions include RA and hematological malignancies such as acute myeloid leukemia, systemic lupus erythematosus, and scleroderma.^[4,7]^ In patients with PG, the prevalence of IBD, RA, and hematological malignancies was approximately 20.2%, 11.8%, and 3.9%, respectively. These comorbidities suggest that PG may share pathophysiological mechanisms with these diseases. Current hypotheses propose that genetic variations found in PG patients, such as mutations in *PSTPIP1*, *MEFV*, and *NOD2*, may be linked to various autoinflammatory conditions.^[8]^ Additionally, abnormal activation of T cells and neutrophils, along with elevated levels of cytokines from the IL-1, IL-36, and IL-17 families, is believed to play a pivotal role in the inflammatory process of PG.^[1]^ In the cohort of 14 patients included in this study, 2 had concurrent IBD and 2 had multiple myeloma. Metabolic-related diseases such as diabetes, hypertension, hyperlipidemia, and cardiovascular diseases are relatively common among these patients. Furthermore, 4 patients presented with anemia, which cannot be ruled out as being related to the prolonged course and chronic debilitating nature of PG.

The diagnosis of PG remains a challenge because of the lack of uniform diagnostic criteria that rely heavily on a diagnosis of exclusion. The first diagnostic criteria for classic ulcerative PG were proposed in 2004 and require the fulfillment of all major criteria and at least 2 minor criteria for diagnosis. The major criterion consisted of rapidly progressing painful necrotic cutaneous ulcers with irregular, violaceous, and undermined borders after other causes of skin ulceration were excluded. The minor criteria included: (1) pathergy or abnormal skin response to minor trauma or the clinical finding of cribriform scarring, (2) associated systemic disease related to PG, (3) histopathological findings (sterile dermal neutrophilia, mixed inflammation, lymphocytic vasculitis), and (4) treatment response (rapid response to systemic corticosteroid therapy).^[9]^ In 2018, a new Delphi consensus was published on the diagnostic criteria for PG, stating that the diagnosis could be made using 1 major and 4 minor criteria. The major criterion was neutrophilic infiltration at the ulcer edge on the biopsy. The 8 minor criteria are as follows: (1) exclusion of infection; (2) positive pathergy test; (3) history of IBD or inflammatory arthritis; (4) pustules, papules, or vesicles evolving into ulcers within 4 days; (5) erythema, undermined borders, and tenderness around the ulcer; (6) multiple ulcers with at least one location on the extensor surface of the lower leg; (7) cribriform or “wrinkled paper” scars at the site of healed ulcers; and (8) reduction in ulcer size within 1 month after treatment with immunosuppressive drugs.^[10]^ The 14 patients included in this study presented with painful, creeping, multiple ulcers surrounded by erythema, histopathology indicative of diffuse dermal inflammation predominantly with neutrophils and lymphocytes, and an effective response to immunosuppressive drugs, such as corticosteroids, aligning with the aforementioned diagnostic criteria and expert consensus.

The therapeutic strategies for PG vary, with the primary goals of controlling inflammation, promoting ulcer healing, managing complications, and addressing any associated comorbidities. For patients with mild disease or those contraindicated for systemic treatment, topical therapies such as ultra-potent corticosteroids, calcineurin inhibitors, dapsone, 5-aminosalicylic acid, and nicotine patches can serve as adjunctive treatments. Systemic therapy for PG typically starts with fast-acting immunosuppressive medications, such as corticosteroids (e.g., prednisone) and cyclosporine, to quickly control inflammation. In cases of severe disease or where traditional treatments are ineffective, biological agents, particularly anti-TNF medications such as infliximab, may be considered. A combination of multiple drugs may be necessary for patients with severe disease or those unresponsive to monotherapy. These include immunomodulatory antibiotics (e.g., dapsone, sulfapyridine, and minocycline), conventional immunosuppressants (e.g., azathioprine, mycophenolate mofetil, and methotrexate), biologics (e.g., anti-TNF, anti-IL-12/IL-23, anti-IL-17, IL-1 receptor antagonists, anti-IL-1β, and anti-IL-6 receptor agents),^[11,12]^ intravenous immunoglobulin, phosphodiesterase 4 inhibitors (e.g., apremilast), and JAK-STAT inhibitors (e.g., tofacitinib and ruxolitinib).^[13]^ Each treatment regimen should be tailored to the individual patient’s condition and response to therapy.^[14–16]^ Proper wound care, which includes meticulous cleaning, dressing, and the potential use of antibiotics, is crucial in the treatment of PG.^[17,18]^ However, it is imperative to avoid surgical debridement to prevent pathergy reactions, which can lead to lesion enlargement. Patients with PG may experience severe pain, necessitating appropriate pain management strategies, including the administration of oral analgesics. The significant impact of PG on a patient’s quality of life underscores the importance of psychosocial support and education as integral components of treatment regimens. Following the healing of PG ulcers, it is essential to establish strategies to prevent recurrence, which may involve maintenance therapy or immediate medical attention at the onset of relapse.^[19]^ In this study, 14 patients were primarily treated with systemic corticosteroids, 3 of whom also received the antibiotic minocycline as an immunosuppressive agent. The overall treatment outcomes were favorable, with patients experiencing a rapid reduction in skin lesions. However, 2 patients exhibited suboptimal responses to corticosteroids, with initial doses of methylprednisolone at 40 mg qd and 60 mg qd. These patients were subsequently treated with mycophenolate mofetil 0.5 bid and cyclophosphamide 0.1 qd, which led to well-controlled skin rashes. These findings suggest that for patients with severe conditions who do not respond adequately to full doses of corticosteroids, the inclusion of immunosuppressive agents can contribute to rapid control of the disease.

In summary, PG is a relatively rare immunologically associated skin disorder characterized by rapidly progressing painful ulcers that significantly impair the quality of life of patients. Our analysis of a small sample size indicated that PG is not uncommon among males in the Chinese population. The management of PG should primarily involve immunosuppression, with systemic corticosteroids providing rapid symptom control in most patients. For those who do not respond well to corticosteroids or where their use is limited, it is imperative to initiate combination therapy with immunosuppressive agents or novel targeted therapies such as biologics or small-molecule drugs at an early stage. Skin lesion care should focus on preventing infection and avoiding surgical debridement. Pain management and symptomatic treatment of complications should also be considered.

### 4.1. Limitations

This study offers important insights into the management of PG; however, further research is necessary to improve our understanding and patient care. The small sample size limits the generalizability of the findings, and the retrospective nature may introduce biases due to reliance on existing medical records. Conducted at a single hospital, the study’s applicability to other settings is also constrained. Moreover, the focus on systemic glucocorticoid treatment overlooks other therapeutic options like biologics or small-molecule drugs. To address these limitations, future studies should involve larger, more diverse populations and explore a wider range of treatments, providing a more comprehensive understanding of the disease and enhancing clinical practice.

## Acknowledgments

We would like to express my heartfelt gratitude to all our colleagues in the dermatology department for their invaluable discussions and unwavering support throughout the process of caring for these patients.

## Author contributions

**Conceptualization:** Rina Su, Shiguang Peng

**Formal analysis:** Rina Su, Shiguang Peng

**Investigation:** Rina Su, Shiguang Peng

**Methodology:** Rina Su, Yaqi Tan, Shiguang Peng

**Writing—original draft:** Rina Su, Shiguang Peng

**Writing—review & editing:** Rina Su, Shiguang Peng

**Resources:** Yaqi Tan, Shiguang Peng

**Supervision:** Shiguang Peng

## References

[1] Maverakis E Marzano AV Le ST. Pyoderma gangrenosum. Nat Rev Dis Primers. 2020;6:81. 33033263 10.1038/s41572-020-0213-x

[2] George C Deroide F Rustin M. Pyoderma gangrenosum - a guide to diagnosis and management. Clin Med (Lond). 2019;19:224–8. 31092515 10.7861/clinmedicine.19-3-224 PMC6542232

[3] Xu A Balgobind A Strunk A Garg A Alloo A. Prevalence estimates for pyoderma gangrenosum in the United States: an age- and sex-adjusted population analysis. J Am Acad Dermatol. 2020;83:425–9. 31400451 10.1016/j.jaad.2019.08.001

[4] Langan SM Groves RW Card TR Gulliford MC. Incidence, mortality, and disease associations of pyoderma gangrenosum in the United Kingdom: a retrospective cohort study. J Invest Dermatol. 2012;132:2166–70. 22534879 10.1038/jid.2012.130

[5] Monari P Moro R Motolese A. Epidemiology of pyoderma gangrenosum: results from an Italian prospective multicentre study. Int Wound J. 2018;15:875–9. 29877043 10.1111/iwj.12939 PMC7949684

[6] Ashchyan HJ Butler DC Nelson CA. The association of age with clinical presentation and comorbidities of pyoderma gangrenosum. JAMA Dermatol. 2018;154:409–13. 29450453 10.1001/jamadermatol.2017.5978 PMC5876860

[7] Kridin K Cohen AD Amber KT. Underlying systemic diseases in pyoderma gangrenosum: a systematic review and meta-analysis. Am J Clin Dermatol. 2018;19:479–87. 29721816 10.1007/s40257-018-0356-7

[8] Marzano AV Borghi A Meroni PL Cugno M. Pyoderma gangrenosum and its syndromic forms: evidence for a link with autoinflammation. Br J Dermatol. 2016;175:882–91. 27106250 10.1111/bjd.14691

[9] Su WP Davis MDP Weenig RH Powell FC Perry HO. Pyoderma gangrenosum: clinicopathologic correlation and proposed diagnostic criteria. Int J Dermatol. 2004;43:790–800. 15533059 10.1111/j.1365-4632.2004.02128.x

[10] Maverakis E Ma C Shinkai K. Diagnostic criteria of ulcerative pyoderma gangrenosum: a Delphi consensus of international experts. JAMA Dermatol. 2018;154:461–6. 29450466 10.1001/jamadermatol.2017.5980

[11] O’Connor C Gallagher C Hollywood A Paul L O'Connell M. Anakinra for recalcitrant pyoderma gangrenosum. Clin Exp Dermatol. 2021;46:1558–60. 34137070 10.1111/ced.14809

[12] Hong JJ Hadeler EK Mosca ML Brownstone ND Bhutani T Liao WJ. Off-label uses of TNF-a inhibitors and IL-12/23 inhibitors in dermatology. Dermatol Online J. 2021;27:1. 10.5070/D3271156085 35130397

[13] Köken Avşar A Demirci Yildirim T Sari I. Tofacitinib therapy for severe pyoderma gangrenosum in a patient with enteropathic arthritis: a case-based review. Rheumatol Int. 2024. doi: 10.1007/s00296-024-05560-1. 10.1007/s00296-024-05560-1 38488863

[14] Maronese CA Pimentel MA Li MM Genovese G Ortega-Loayza AG Marzano AV. Pyoderma gangrenosum: an updated literature review on established and emerging pharmacological treatments. Am J Clin Dermatol. 2022;23:615–34. 35606650 10.1007/s40257-022-00699-8 PMC9464730

[15] Dissemond J Marzano AV Hampton PJ Ortega-Loayza AG. Pyoderma gangrenosum: treatment options. Drugs. 2023;83:1255–67. 37610614 10.1007/s40265-023-01931-3 PMC10511384

[16] Yamanaka K. New treatment of pyoderma gangrenosum and hidradenitis suppurativa: a review. J Dermatol. 2024;51:172–9. 38009911 10.1111/1346-8138.17031 PMC11483966

[17] Croitoru D Naderi-Azad S Sachdeva M Piguet V Alavi A. A wound care specialist’s approach to pyoderma gangrenosum. Adv Wound Care (New Rochelle). 2020;9:686–94. 32320358 10.1089/wound.2020.1168 PMC7698649

[18] Strunck JL Cutler B Latour E Seminario-Vidal L Ortega-Loayza AG. Wound care dressings for pyoderma gangrenosum. J Am Acad Dermatol. 2022;86:458–60. 34600958 10.1016/j.jaad.2021.09.053

[19] Alavi A French LE Davis MD Brassard A Kirsner RS. Pyoderma gangrenosum: an update on pathophysiology, diagnosis and treatment. Am J Clin Dermatol. 2017;18:355–72. 28224502 10.1007/s40257-017-0251-7

